# Intercellular Adhesion Molecule 1 (ICAM-1): An Inflammatory Regulator with Potential Implications in Ferroptosis and Parkinson’s Disease

**DOI:** 10.3390/cells13181554

**Published:** 2024-09-15

**Authors:** Matthew R. Miller, Harold E. Landis, Robert E. Miller, Yousef Tizabi

**Affiliations:** 1 NutriGenetic Research Institute, Ephrata, PA 17522, USA; 2 Integrative Medicine Fellow, University of Arizona College of Medicine, Tucson, AZ 85724, USA; 3 Department of Pharmacology, Howard University College of Medicine, 520 W Street NW, Washington, DC 20059, USA

**Keywords:** ICAM-1, Parkinson’s disease, ferroptosis, glial cells, T cells, neuroinflammation

## Abstract

Intercellular adhesion molecule 1 (ICAM-1/CD54), a transmembrane glycoprotein, has been considered as one of the most important adhesion molecules during leukocyte recruitment. It is encoded by the *ICAM1* gene and plays a central role in inflammation. Its crucial role in many inflammatory diseases such as ulcerative colitis and rheumatoid arthritis are well established. Given that neuroinflammation, underscored by microglial activation, is a key element in neurodegenerative diseases such as Parkinson’s disease (PD), we investigated whether ICAM-1 has a role in this progressive neurological condition and, if so, to elucidate the underpinning mechanisms. Specifically, we were interested in the potential interaction between ICAM-1, glial cells, and ferroptosis, an iron-dependent form of cell death that has recently been implicated in PD. We conclude that there exist direct and indirect (via glial cells and T cells) influences of ICAM-1 on ferroptosis and that further elucidation of these interactions can suggest novel intervention for this devastating disease.

## 1. Introduction

Intercellular adhesion molecule 1 (ICAM-1/CD54) is a transmembrane glycoprotein that was discovered in the 1980s and was identified as a ligand of the β_2_ integrin lymphocyte function-associated antigen (LFA)-1 (CD11a/CD18), and as an important switch to initiate a key adhesion pathway [1,2]. Since then, its critical role in inflammatory responses and a plethora of inflammatory diseases has been verified [3,4,5,6,7,8,9].

Although neurodegenerative diseases in general, and Parkinson’s disease (PD) in particular, are triggered and/or exacerbated by neuroinflammatory mediators, an association between ICAM-1 and PD has not been adequately studied. Justification for such pursuit is supported by several premises. First, there is a well-established involvement of neuroinflammation in PD [10,11,12,13]. Second, the co-morbid presentation of depression with PD is extensively documented [14,15,16], and the involvement of ICAM-1 in late-life depression has been verified [17]. Moreover, the presence of ICAM-1 in reactive astrocytes was identified in patients with PD as well as in 1-methyl-4-phenyl-1,2,3,6-tetrahydropyridine (MPTP)-treated monkeys, a nonhuman primate model of PD [18]. Therefore, the aim of the current review is to provide the mechanistic implications of ICAM-1 in PD, specifically in relation to glial cell-mediated neuroinflammation underscored by ferroptosis, a recently implicated pathway in PD pathology, as well as T cell reactivity. Thus, following brief descriptions of ICAM-1, PD, glial cells, T cells, and ferroptosis, we seek to provide convincing evidence on a causal relationship between them, with the hope of identifying novel targets for the treatment of PD.

### 1.1. ICAM-1

The well-known function of ICAM-1 involves leukocyte extravasation and has been described as one of the most important adhesion molecules during leukocyte recruitment [19,20,21]. Specifically, the expression of *ICAM1*, located on chromosome 19, is induced in endothelial cells by a variety of cytokines and inflammatory mediators, including tumor-necrosis factor alpha (TNF-α), nuclear factor kappa B (Nf-kB), interferon gamma (IFN-γ), interleukin-1 beta (IL-1β), IL-6, as well as hydrogen peroxide (H_2_O_2_) and NADPH oxidase (NOX) enzyme activity [1,22,23,24,25,26,27]. ICAM-1 is expressed in the plasma membrane and binds to the β_2_ integrins LFA-1 and macrophage antigen 1 (MAC-1/CD11b/CD18) expressed by leukocytes [28,29,30]. ICAM-1-LFA-1/MAC-1 binding mediates leukocyte rolling, crawling, adhesion, and the passage of blood cells through the intact walls of the capillaries (diapedesis), often accompanied by inflammation during extravasation, the process by which leukocytes move out of the circulatory system to the site of tissue damage or infection [30]. Upon binding to LFA-1/MAC-1, ICAM-1 induces the dissociation of junction proteins with adjacent endothelial cells, cytoskeletal rearrangement, and endothelial nitric oxide synthase (eNOS) activity, enabling transendothelial leukocyte migration [9,30,31,32]. However, as discussed below, the implications of ICAM-1 extend beyond the transmigration of leukocytes.

ICAM-1 is expressed in neurons and immune, endothelial, and epithelial cells, among others, albeit at low levels of expression during basal conditions [33,34,35]. ICAM-1 has several notable ligands, including fibrinogen, mucin 1 (MUC1), cluster of differentiation 43 (CD43), hyaluronan, rhinoviruses, and plasmodium falciparum [36,37,38,39,40,41]. Moreover, ICAM-1 is involved in a myriad of physiological processes, such as T cell regulation (discussed below), macrophage polarization, cellular migration, reactive oxygen species (ROS) production, and cancer development and metastasis [7,26,42,43,44]. Aldosterone and angiotensin II have been found to induce atherosclerosis and hypertension, respectively, via ICAM-1-dependent mechanisms in experimental models [45,46,47]. The infusion of angiotensin II also increases ICAM-1 in human subjects [45]. The role of ICAM-1 in atherosclerosis and cardiovascular disorders has been extensively evaluated and verified [8,46,47,48]. The adhesion molecule appears to have a fundamental role in intestinal and blood–brain barrier (BBB) permeability, and neuroinflammation [49,50,51,52,53]. In addition to inflammation remediation, ICAM-1 also plays a role in wound healing and efferocytosis or the clearance of apoptotic cells [7,54,55].

**Figure 1:**
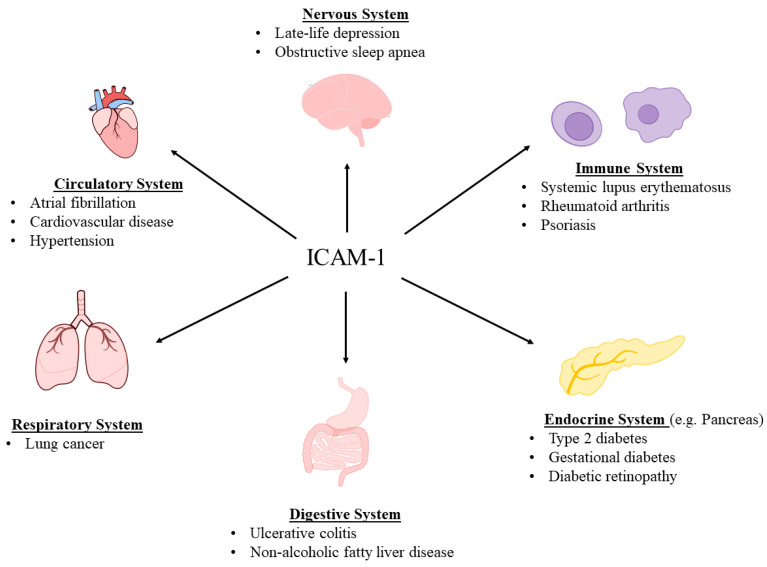
ICAM-1 has been implicated in and associated with an array of diseases.

As a transmembrane glycoprotein, ICAM-1 is expressed in the plasma membrane and extends into the cytoplasm and onto the cell surface, enabling the participation in signal transduction, interactions with cytoskeletal structures, and ligand binding [7,30,56]. Nevertheless, ICAM-1 can be enzymatically cleaved from the cell surface to circulate freely as a form of the protein known as soluble ICAM-1 (sICAM-1) [57,58]. Specifically, a disintegrin and metalloproteinase 10 (ADAM10), ADAM17, matrix metalloproteinase 2 (MMP-2), and MMP-9 have been found to cleave membrane-bound ICAM-1 [59,60,61,62]. The role of leukocyte elastase and cathepsin G may be particularly relevant regarding cleaving ICAM-1 isoforms that arise due to alternative splicing [63,64]. Furthermore, sICAM-1 can be generated because of alternative splicing, in which case it lacks transmembrane and cytoplasmic domains [30,65]. In vitro sICAM-1 concentrations have been found to quantitatively relate to cell surface ICAM-1 expression [58,66]. Conversely, alternative splicing, protease activity, and ICAM-1 ectodomain shedding would have the potential to impact this relationship and should be considered during in vivo analyses [30,65,67]. sICAM-1 concentrations have been reported to range from 100 to 450 ng/mL in the serum of the general population [4,68]. Increased sICAM-1 levels have been associated with an array of conditions, notably, endometriosis, systemic lupus erythematosus, rheumatoid arthritis, psoriasis, obstructive sleep apnea, non-alcoholic fatty liver disease, lung cancer, atrial fibrillation, obesity, type 2 diabetes, diabetic retinopathy, gestational diabetes mellitus, and late-life depression [17,69,70,71,72,73,74,75,76,77,78,79,80,81,82]. More recently, its crucial role in ulcerative colitis (an inflammatory bowel disease), where higher levels of ICAM-1 were associated with worse prognosis, was revealed [83] (Figure 1). 

Thus, ICAM-1 has central yet diverse roles in inflammation and has an important role in the initiation of inflammatory responses. Nonetheless, the entirety of these implications has yet to be elucidated in specific phenotypes and/or disease states. Of particular importance and unexplored implication is to what extent and through what mechanisms ICAM-1 may be involved in PD pathology.

### 1.2. Parkinson’s Disease (PD)

PD is a progressive neurodegenerative disease marked by the gradual deterioration of dopaminergic (DAergic) neurons in the substantia nigra pars compacta (SNpc), the accumulation of misfolded α-synuclein proteins as Lewy bodies, and the dysregulation of glial cells (discussed below). Although both genetic and environmental influences are known risk factors, most PD cases are sporadic or what is commonly referred to as idiopathic, meaning no known cause. PD is characterized by motor and non-motor symptoms. The motor symptoms include resting tremor, bradykinesia, rigidity or inflexibility, dystonia, and postural and walking abnormalities [11,84]. Freezing of gait is also a common feature. Non-motor symptoms, which often precede the motor symptoms, include partial or total loss of smell (anosmia), mood disorders (e.g., depression), excessive sweating, hypotension, fatigue, cognitive impairment, inability to produce facial expressions or recognize other’s verbal and nonverbal cues, sleep perturbations (e.g., insomnia/hypersomnia), gastrointestinal problems (e.g., difficulty in swallowing, constipation, and nausea), and urinary and sexual dysfunction [11,85]. 

Since DA loss is the primary underlying cause, therapeutic interventions are focused on replacing this neurotransmitter or its function. This is primarily achieved by the administration of L-dopa, considered the gold standard, plus drugs that interfere with DA breakdown, such as monoamine oxidase or catechol-o-methyltransferase inhibitors (e.g., selegiline/rasagiline and tolcapone/entacapone, respectively) and/or newer non-ergot DA agonists, such as pramipexole, ropinirole, rotigotine, and apomorphine [10,86]. Carbidopa is given in conjunction with L-dopa to prevent its peripheral breakdown. All of these drugs may provide remarkable symptomatic relief, yet none of them address the progression of neurodegeneration. Moreover, L-dopa, the most efficacious drug, not only loses its efficacy over months or years but may induce severe dyskinesia, which may be worse than the initial tremors [10,87]. Hence, more efficacious interventions without such adverse effects are urgently needed [11,88].

Neuroinflammation and oxidative stress have been extensively implicated in PD [11,89,90,91,92]. Oxidative stress (OS) occurs due to an imbalance of oxidants and antioxidant capacity, which can lead to oxdiant-induced damage to DNA, proteins, and lipids [11]. Within the context of PD, oxidants may originate from a variety of sources, which includes, but is not limited to, mitochondrial dysfunction, DA metabolism, and glial cells [11,89,90,91,92]. Oxidative stress and ROS can induce the activation of immune cells and immune responses, and ultimately inflammation. However, inflammation and OS have a bidirectional relationship, which can contribute to a vicious and reciprical cycle in PD [11,89,90,91,92]. Mitochdondrial damage and NOX also have roles in this process [90,91,92]. An emphasis of this review is the elucidation of how ICAM-1 interacts with the intermediaries of OS or inflammation and how it might provide a therapeutic target in PD. 

### 1.3. Glial Cells 

Glial cells were first identified in the mid-19th century and were referred to as neuroglia (neuro-glue), since they were thought to provide merely structural support for the neurons. However, it is now known that glial cells carry a variety of crucial functions, not only as structural support for neurons [93,94,95], but also in myelination [96,97], the control of energetics and metabolism [95,98,99], the formation of the BBB [100,101], the development and remodeling of synapses [102,103], the control of the fluid/electrolyte homeostasis [104], the regulation of neurotransmitters [105,106], neuroendocrine function [107], detoxification [108,109], and innate immunity response [110,111]. It is not surprising, therefore, that their disruption or dysregulation may lead to neuropsychiatric and neurodegenerative diseases [13,97,112,113,114,115,116]. By the same token, they may present novel targets in neurological diseases [117]. Indeed, it has been suggested that the manipulation of the nicotinic cholinergic receptors (nAChRs) in these cells may be a viable target for intervention in PD [13], mood disorders, and even drug addiction [118].

Four main types of glial cells include microglia, astrocytes, oligodendrocytes, and synantocytes or NG2 cells. Below, following a brief description of each, we specifically concentrate on their interactions with ICAM-1 vis-a-vis neuroinflammatory response.

#### 1.3.1. Microglia

Microglia, representing 10–15% of all of the central nervous system (CNS) cells, cover a significant volume of the adult brain parenchyma. These cells, through rapid movements of their fine filopodia, constantly survey the environment and react quickly to any kind of insult. They share the same origin as peripheral macrophages but are considered the resident immune cells of the CNS [119,120]. By regulating neurogenesis, the formation and elimination of neuronal synapses, mediating T cell infiltration into the brain, and, most importantly, eliminating pathogens and cell residues, they play a vital role in maintaining brain homeostasis [121]. On the other hand, if overactivated, microglia can cause neuroinflammation, leading to neuronal damage or death, and neuropsychiatric and/or neurodegenerative diseases, including PD [122,123,124,125]. A major culprit in microglial overactivation is persistent stress causing the release of proinflammatory mediators such as IL-1β and IL-6 [126,127,128].

It is of relevance to note that, based on their activation state, different microglial subtypes were described previously. Hence, the M1 microglia was associated with a proinflammatory state and the M2 with an opposite or anti-inflammatory state [121,129]. However, emerging evidence suggests that differences in microglia functions are due to their inherent properties, and that the subtypes should be categorized based on their function and avoid the use of the M1 or M2 state as such [117,130,131].

Microglia express various receptors, including the calcium-sensing receptor (CASR), low-density lipoprotein receptor-related protein 1 (LRP1), triggering receptor expressed on myeloid cells-2 (TREM2), nAChRs, and Toll-like receptors such as TLR2 and TLR4 [13]. TLRs are a well-characterized family of pattern-recognition receptors (PRRs) that initiate the innate immune response by sensing the endogenous debris or pathogens. Because of their significant role in neurodegenerative diseases, TLRs are investigated intensely as potential therapeutic targets in such diseases [121,132,133,134].

#### 1.3.2. Astroglia

Due to their star-like shape, these cells were named astroglia or astrocytes [135] and may constitute anywhere between 17 and 61% of the total brain cells, depending on the area. Astrocytes also play a crucial role in maintaining neuronal integrity and function, as they provide nutrients, monitor and regulate pH homeostasis, remove waste, and are a key constituent of the BBB [135,136].

Astrocytes contain both the glial-derived neurotrophic factor (GDNF), that provides trophic support to neuronal cells including DAergic neurons [137], and glial fibrillary astrocytic protein (GFAP), which is a key protein responsible for maintaining astrocyte strength and the BBB. The GFAP is commonly used as a marker for astrocyte identification [138] and may serve as a biomarker for brain and spinal cord disorders [139,140,141,142]. These glial cells also express brain-derived neurotrophic factor (BDNF) and the highest amount of taurine, a free amino acid with antioxidant and anti-inflammatory properties that is required for optimal postnatal brain development [143]. More recently it was reported that astrocytes are the necessary source of TNF-α for the mediation of homeostatic synaptic plasticity [144].

Astrocytes in conjunction with microglia provide the first line of defense against insults. Here, also, the overstimulation of the proinflammatory signals may synergistically contribute to neuronal dysregulation and ensuing neuropsychiatric/neurodegenerative diseases [145,146,147]. Moreover, the elucidation of the intimate interaction between astrocytes and microglia, as well as astrocytes and neurons, referred to as crosstalk, could provide novel intervention in such diseases [137,148,149,150]. 

#### 1.3.3. Oligodendrocytes 

Oligodendrocytes (OLs), representing 75% of all glial cells, are the major source of myelination in the CNS [151]. In addition to axonal myelination, OLs have other crucial functions, such as providing the metabolic and trophic supply by the secretion of GDNF and BDNF, controlling the extracellular potassium concentration, and modulating axonal growth [151,152]. They also express TLRs, which are important in myelin formation [96,153,154]. It is not surprising, therefore, that the dysregulation of these glial cells could lead to a variety of neurological diseases, including PD (discussed in more detail below). 

#### 1.3.4. Synantocytes (NG2 Cells) 

The fourth subset of major glial cells in the CNS are synantocytes, which are also referred to as neuron glial 2 or nerve/glial antigen 2 (NG2) cells, and oligodendrocyte precursor cells (OPCs). NG2 cells are expressed in both white and gray matter areas, and can keep proliferating in the adult brain [151,155,156]. In addition to being OL progenitors, NG2 cells can also transform to astrocytes and neurons [151,155,156]. They have been implicated in a variety of neurological disorders, including multiple sclerosis, Alzheimer’s disease (AD), epilepsy, traumatic brain injury, acute ischemic stroke, neurovascular unit formation during development, glioma, and experimental autoimmune encephalomyelitis (EAE), a disease associated with increased BBB permeability and neuroinflammation [157,158,159,160,161]. Moreover, their communication and influence on neurons renders them a potential therapeutic target in many diseases, including PD [162,163], as discussed in more detail below.

### 1.4. ICAM-1—Glial Cells 

#### 1.4.1. ICAM-1—Microglia

Microglia express ICAM-1 and constitutively express LFA-1 and Mac-1, which enables various direct interactions between ICAM-1 and microglia within multiple contexts [164,165]. Notably, ICAM-1 has a role in the activation of microglia [166,167,168]. Activated microglia, in turn, secrete TNF-α, which induces the expression of ICAM-1 in vascular endothelial cells and facilitates leukocyte infiltration [23,169,170]. ICAM-1 may also indirectly activate microglia. This occurs due to the vascular endothelial expression of ICAM-1, which promotes the transendothelial migration of leukocytes and their infiltration into the CNS, resulting in microglial activation [171,172,173,174]. Interestingly, leukocytes that have infiltrated into the CNS may adhere to microglia [171,175]. Thus, there is a positive feedback loop between ICAM-1 and microglia [166,167,168,171,172,173,174].

#### 1.4.2. ICAM-1—Astroglia

Astrocytes also contain ICAM-1, the expression of which is increased by TNF-α, IL-1β, and IFN-γ [176,177,178,179,180]. ICAM-1, in turn, may cause the release of inflammatory cytokines, including TNF-α in astrocytes [181,182]. The ICAM-1 activation of astrocytes may also be brought indirectly via fibrinogen, which is induced in various neuroinflammatory states and binds to ICAM-1 [183,184]. Fibrinogen-activated astrocytes further enhance ICAM-1 expression and promote the production of NO and ROS, leading to neuronal death [183,184]. Curiously, ROS may induce astrocytic ICAM-1 production in an Nf-kB-dependent mechanism [185,186]. Therefore, here, also, there appears to exist a positive feedback loop between astrocytes and ICAM-1 [179,181,183,184,185,186].

#### 1.4.3. ICAM-1—Oligodendrocytes

The enhanced expression of ICAM-1 in OLs during inflammatory conditions is postulated to be a defense mechanism in response to immunogenic insult [177,187]. The direct contact of OLs with T cells has been suggested to induce OL damage, and anti-ICAM-1 antibodies were found to inhibit Th1 cell contact with OLs [187]. Due to the role of OLs in myelination, T cell-induced damage in these cells may contribute to neurodegeneration, as seen in EAE [187]. The inhibitory action of anti-ICAM-1 antibodies on EAE in vivo has been shown in animal models, including marmoset monkeys [188,189]. As discussed for microglia, ICAM-1 has a role in T cell infiltration into the CNS, which again provides an indirect mechanism for ICAM-1 to influence OL homeostasis [173,177,187].

#### 1.4.4. ICAM-1—NG2 Cells

The maturation of NG2 cells is inhibited by proinflammatory cytokines [190,191,192]. Moreover, microglia can influence NG2 cell proliferation, differentiation, migration, and apoptosis, while NG2 cells can regulate microglia homeostasis and activation [193]. This suggests an indirect interaction between ICAM-1 and NG2 cells. While recent findings implicate NG2 cells in the initiation of neuroinflammation via the activation of immunogenic cells, the NG2 protein appears to be a negative regulator of ICAM-1 expression in pericytes and two different glioblastoma cell lines [194,195,196].

### 1.5. ICAM-1—Glial Cells—PD

Neuroinflammation has been extensively implicated in the pathophysiology of PD, with significant contributions from glial cells [197,198]. The role of glial cells in neuroinflammation and PD is supported by a recent meta-analysis reporting increased cerebral spinal fluid (CSF) concentrations of TNF-α, IL-6, IL-1β, nitric oxide (NO), chemokine ligand 2 (CCL2), and c-reactive protein (CRP) in individuals with this disease [199]. Despite the protective roles of transiently activated microglia, the chronic activation of microglia has been widely hypothesized to be involved in PD [200]. Specifically, inflammatory microglia phenotypes have been found in experimental models as well as in the SN of PD patients [200,201]. Cytokines associated with inflammatory phenotypes of microglia include TNF-α, IL-6, IL-1β, and IFN-γ, all of which induce ICAM-1 expression [1,22,23,199,202]. The role of microglia in PD is also supported by a postmortem analysis that found an increase in activated microglia, and its expression of ICAM-1, LFA-1, TNF-α, and IL-6, in the SN and various other regions of the brain in PD patients [203]. 

While microglia have a clear role in PD-associated neuroinflammation, they may also induce a neurotoxic and reactive phenotype of astrocytes, often referred to as the A1 phenotype, which can exacerbate PD pathology [204,205,206]. Furthermore, astrocytes have a role in the removal of dysfunctional proteins such as α-synuclein. Microglial–astrocyte interactions may also facilitate α-synuclein removal, the accumulation of which may increase ICAM-1 and IL-6 expression in astrocytes [206,207,208,209]. Moreover, increased concentrations of astrocytes and microglia, infiltration of leukocytes, and increased expression of ICAM-1 and LFA-1 were found in the SN of PD patients [18].

Abnormal and decreased myelin contents have been associated with PD symptoms [210,211]. In PD patients, 80% of connections originating from the basal ganglia displayed decreased myelin content [212]. Moreover, OLs, which are major contributors to myelination, have been found to be decreased in idiopathic PD [213]. In addition, α-synuclein transfer from neurons to OLs may exacerbate PD pathology [214,215]. Given that the OL expression of ICAM-1 is enhanced during inflammatory conditions, the modulation of this adhesion molecule may provide a novel target in PD [177,187]. 

Altogether, the above provides a strong connection between ICAM-1, glial cells, and PD.

### 1.6. ICAM-1—T Cells

Leukocytes in the circulatory system are recruited to sites of inflammation via various inflammatory signaling molecules, such as cytokines and chemokines. As mentioned earlier, once leukocytes reach the site of inflammation, they often extravasate and undergo diapedesis, that is, they pass through capillary walls, a process mediated by adhesion molecules. T cells also undergo a similar process as they express LFA-1 and interact with ICAM-1 to facilitate their endothelial transmigration [216,217]. ICAM-1 not only facilitates T cell transmigration [216,218], but also facilitates their activation [219,220]. In addition, it plays an important role in enabling T cell interactions with other leukocytes [219,220,221]. 

### 1.7. ICAM-1—T Cells—PD

T cells have diverse roles in PD and appear to be influenced by DA [222]. Moreover, peripheral concentrations of T cell subpopulations are generally heterogenous and are dependent on a variety of patient characteristics, such as sex, age, and disease severity and duration [223,224]. Specifically, PD patients present with increased Th1 and Th17 cells and decreased Th2 and regulatory T cells (Tregs) [224], and some PD patients possess α-synuclein-specific T cells [225,226,227]. 

Under inflammatory conditions, endothelial cells in the CNS express various proteins and adhesion molecules, including ICAM-1, which facilitate the migration and infiltration of immune cells and antibodies [225,228]. T cell infiltration into the CNS of PD patients is supported by numerous animal studies, including nonhuman primates, as well as postmortem human studies [229,230,231,232,233,234]. For example, it was shown that ICAM-1 and LFA-1 expression were increased in endothelial and T cells, respectively, and that the administration of ICAM-1 or LFA-1 antibodies reduced immunological and behavioral changes in MPTP-treated mice [231]. Furthermore, contact between CD8 T cells and dopaminergic cells was observed in postmortem PD patients [231,233]. The ICAM-1/LFA-1 axis has also been shown to mediate the Th17-induced death of dopaminergic neurons [35]. Thus, it can be asserted that ICAM-1 interaction with T cells is part of PD pathology. As discussed, increased ICAM-1 expression in PD may be most relevant in endothelial cells and the BBB, and in glial cells [18,203,235,236,237,238]. Regarding circulating sICAM-1 concentrations, although decreased *ICAM1* gene expression has been detected in PD patients [239], increased sICAM-1 levels have also been noted in the sera, plasma, and CSF of such patients [231,240,241,242]. Therefore, it remains to be determined how the gene expression of *ICAM1* may translate into protein production in PD. 

## 2. Fe—Ferroptosis

Ferroptosis is an iron-dependent form of regulated cell death that is unique relative to other mechanisms of cell death [243,244]. While the term and concept of ferroptosis was introduced in 2012, the central role of iron in non-apoptotic cell death emerged several years prior, in 2008 [244,245]. Ferroptosis has since been implicated in an array of conditions, including diseases of the liver, kidney, intestines, lungs, heart, blood cells, and nervous systems, among others [246,247,248]. Iron, as Fe^2+^, reacts with hydrogen peroxide (H_2_O_2_) and generates a hydroxyl radical (^•^OH), which induces lipid peroxidation, known as the Fenton Reaction (Fe^2+^ + H_2_O_2_ → Fe^3+^ + ^•^OH + OH^−^) [244]. This reaction and the hydroxyl radical ultimately lead to the peroxidation of cellular membrane lipids and cell death (ferroptosis) [244,246,249]. Iron can also lead to the generation of alkoxyl radicals via the reaction with lipid hydroperoxides and the activation of arachidonate lipoxygenase (ALOX) enzymes [246,249]. ALOX enzymes oxygenate polyunsaturated fatty acids (PUFAs), leading to the generation of lipid hydroperoxides, and subsequently malondialdehyde (MDA) and 4-hydroxynonenal. Due to the role of PUFAs in ferroptosis, the PUFA-synthesizing enzymes ACSL4 and LPCAT3 have also been implicated in this process [249].

H_2_O_2_ can originate from a variety of sources, including the reduction of superoxide (O_2_^−^) via superoxide dismutase (SOD) enzymes [250]. The metabolism of DA via monoamine oxidase B also produces H_2_O_2_ [251]. The mitochondria are a major source of superoxide, as electrons can escape from the electron transport chain and react with oxygen [250]. NOX enzymes utilize NADPH as an electron donor to generate superoxide and are also a major source of this free radical [249,250]. Following the reduction of superoxide via SOD, the catalase enzyme can catalyze the reduction of two hydrogen peroxide molecules to water and diatomic oxygen [250]. However, as alluded to earlier, in the presence of iron, hydrogen peroxide can participate in the Fenton Reaction and contribute to the synthesis of the hydroxyl radical, which is a highly potent oxidant [244]. 

Ferroptotic cells have several key features, including abnormal mitochondria. While iron has a central role, the lipid metabolism and glutathione homeostasis are also regulators of ferroptosis. Likewise, glutathione peroxidase 4 (GPX4) is a major regulator of ferroptosis [244]. GPX4 utilizes glutathione (GSH) to reduce peroxidized phospholipids and cholesterol [246]. Due to the importance of glutathione, the cystine–glutamate antiporter xCT, also known as SLC7A11, has a role in mediating ferroptosis. Additionally, SLC7A11 transports cystine into the cytoplasm while transporting glutamate into the extracellular space. In an NADPH-dependent mechanism, cystine is then converted into cysteine, which is a rate-limiting amino acid in the synthesis of glutathione. Notably, erastin, a small-molecule compound, can induce ferroptosis by inhibiting SLC7A11 [252]. GPX4 and SLC11A7 expression is regulated by the transcription factor known as nuclear factor erythroid 2-related factor 2 (Nrf2), which is sequestered and controlled by KEAP1, and binds to the antioxidant response element (ARE) [252,253]. As a result, Nrf2 is an important mediator of ferroptosis and regulates the expression of additional ferroptosis-related genes, such as glutathione synthetase, ferroportin 1 (*FPN1*), heme oxygenase 1 (*HO-1*), transferrin receptors (*TFRC*), and ferritin heavy chain 1 (*FTH1*) [253]. Conversely, the transcription factor Bach1, also known as the BTB domain and CNC homolog 1, represses the expression of several Nrf2-regulated genes, and thus can induce ferroptosis by reducing the expression of glutathione and iron metabolism-related genes [252,253,254,255]. It has also recently been revealed that a specific form of autophagy, known as ferritinophagy, has a notable role in ferroptosis, as it regulates the degradation of ferritin, an intracellular protein that stores and releases iron in a controlled fashion. Hence, ferritinophagy may be considered as a new player in maintaining iron homeostasis [250,253].

### 2.1. Ferroptosis—PD

The role of ferroptosis in the pathophysiology of PD has been extensively discussed and is supported by a wide range of data [85]. Iron can accumulate in the SN of PD patients, leading to the death of dopaminergic neurons [85,256]. PD has also been associated with lipid peroxidation, aberrant iron metabolism, decreased GSH, and ROS production, all of which are reflected in differences in gene expression in the SN of PD patients [85,251,257,258,259]. Specifically, differences in the expression of ferroptosis-related genes have been observed in dopaminergic and non-dopaminergic neurons, microglia, astrocytes, OLs, NG2 cells, and endothelial cells/pericytes of PD patients [259]. 

Interestingly, α-synuclein also has a role in the iron metabolism and PUFA synthesis, as it induces lipid peroxidation and increases the risk of ferroptosis in dopaminergic neurons [85,246,260,261,262]. Iron may also enhance the oxidation of DA, an unstable neurotransmitter, leading to the formation of 6-hydroxydopamine (6-OHDA) and DA quinone (DAQ) [263,264]. DAQ, in turn, may enhance neuron susceptibility to ferroptosis by facilitating the degradation of GPX4 [264,265]. It has also been suggested that Fe^3+^ may be reduced by lipid hydroperoxides, creating an iron–DA complex that produces 6-OHDA and hydroxyl radicals [263,266]. 6-OHDA has been found to increase the concentrations of free iron via releasing it from ferritin, and ultimately may create a vicious cycle of free radical production. This toxic consequence is further enhanced by the metabolism of 6-OHDA leading to H_2_O_2_ generation [263].

Ferroptosis may also have a role in BBB disruption and dysfunction [85,267]. Specifically, increased iron, lipid peroxidation, and decreased antioxidant concentrations have been found in the BBB of PD patients [267]. BBB impairment, which may also involve α-synuclein, has been observed in PD patients [268,269,270,271] and involves disrupted tight junction proteins and adhesion molecules, contributing to the pathophysiology of the disease [267,270].

### 2.2. Glial Cells—Ferroptosis

Glial cells have complex and multifaceted interactions with the iron metabolism and ferroptosis. They can be a direct source of iron in the CNS, as they contain ferritin, the concentration of which is increased during aging and pathological conditions [263]. Glial cells can also indirectly facilitate the influx of iron and inhibit its efflux across the BBB via secreting ceruloplasmin and hepcidin, respectively [263,272]. Hepcidin, a peptide hormone produced in the liver, plays a crucial role in iron homeostasis [85]. Glial cells may also facilitate iron accumulation in the CNS via the cytokine-induced regulation of iron transporters [273].

Since glial cells can regulate iron homeostasis in the CNS and are involved in the induction of ferroptosis, they may have critical roles in neurodegenerative processes [274]. Specifically, activated astrocytes may induce neuronal ferroptosis via secreting the CXCL3R ligand CXCL10 and decreasing the expression of SLC7A11 [275]. On the other hand, in a BDNF- and Nrf2-dependent mechanism, astrocytes may protect dopaminergic neurons from ferroptosis [276,277]. A similar scenario exists for microglia, in which lipopolysaccharide (LPS)-activated microglia may protect neurons against glutamate-induced ferroptosis [278]. The complexity of these interactions is further underscored by the findings that glial cells themselves can undergo ferroptosis, and hence contribute to neurodegeneration [279]. While NG2 cells are particularly prone to ferroptosis, OLs, with their greatest concentrations of iron in the CNS, may protect themselves against ferroptosis by secreting ferritin heavy chain [246,277,280,281].

Additionally, ferroptosis may activate glial cells by releasing damage-associated molecular patterns (DAMPs) [253,282,283,284]. DAMPs are molecules that are released from damaged or dying cells and are considered a component of the innate immune response [253,282]. Glial cells also express PRRs, such as TLRs, that can recognize and activate DAMPs, and hence contribute to neurodegeneration [283,284]. It is, however, important to note that the DAMP-mediated activation of microglia may have neuroprotective effects in some instances [284]. This is consistent with the concept that the acute activation of microglia can have neuroprotective properties, whereas chronic activation can lead to neurodegeneration [200,201].

### 2.3. T Cells—Ferroptosis

In tumor cells, T cells can induce ferroptosis via IFN-γ-mediated SLC7A11 inhibition and ACSL4 activation [285,286,287,288]. This has emerged as an important innate antitumor immune response [285,289]. T cells have also the potential to contribute to neuronal ferroptosis by increasing the expression of transferrin receptor 1 (TfR1) in neurons [290]. Moreover, the interaction between T cells and ferroptosis is reciprocal, as neuronal ferroptosis can activate T cells [291,292], and T cells themselves can undergo ferroptosis [289]. However, it appears that ferroptosis may be less immunogenic than other forms of cell death [244,246]. 

### 2.4. ICAM-1—Ferroptosis

To our knowledge, the interactions between ICAM-1 and ferroptosis in various contexts have not been adequately addressed. Direct bidirectional interactions between ferroptosis and ICAM-1 is suggested by multiple experimental analyses. For example, in an in vivo contusion spinal cord injury model, the ferroptosis inhibitor SRS 16-86 decreased ICAM-1 expression, among other changes in protein and cytokine expression [293]. Similar results were found in a diabetic neuropathy rat model, in which SRS 16-86 reduced ICAM-1 as well as IL-1β and TNF-α [294]. Likewise, ferrostatin-1 (Fer-1), a ferroptosis inhibitor, inhibited oxidized low-density-lipoprotein-induced ICAM-1 expression in endothelial cells [295,296,297]. Moreover, erastin, a ferroptosis inducer, has been found to increase ICAM-1 expression and activate endothelial transmigration [298]. Nonetheless, further verifications of the direct interactions between ICAM-1 and ferroptosis in other models and contexts are required.

Hydrogen peroxide, lipid peroxides, and ROS, which have a central role in ferroptosis, also appear to have a role in ICAM-1 expression [23,26,236,299,300,301,302]. Specifically, hydrogen peroxide has been found to increase ICAM-1 expression in endothelial cells [23,299,300]. It is important, however, to note that some analyses have failed to find the H_2_O_2_-induced expression of ICAM-1 in endothelial cells [169,303], likely due to methodological differences [23,169,299,300,303]. Nevertheless, H_2_O_2_ appears to increase ICAM-1 expression via the AP-1 and Ets cis-regulatory elements in the *ICAM1* gene promoter [23,299]. H_2_O_2_ has also been shown to have a role in the post-translational modification of ICAM-1 [304,305,306]. Additionally, plasma with elevated lipid peroxides obtained from women with severe pre-eclampsia increased ICAM-1 expression in human umbilical cord endothelial cells [302]. 

ICAM-1 was suggested to have a regulatory role in ferroptosis in a recent study in which the administration of recombinant ICAM-1 (rICAM-1) increased intracellular ROS and Fe^2+^, and decreased GPX4 and SLC7A11 expression in LPS-stimulated macrophages and human umbilical cord endothelial cells, likely mediated by *PTGS2*. Moreover, the inhibition of PTGS2 inhibited the impact of rICAM-1 on ferroptosis-related parameters, suggesting that PTGS2 has a mechanistic role in this interaction [307]. However, further elucidation of the mechanism of action of ICAM-1 in its various roles is warranted.

### 2.5. ICAM-1—Glial Cells—T Cells—Ferroptosis—PD

In PD, dopamine oxidation and mitochondrial dysfunction are widely considered to be underlying characteristics of the disease. Dopamine oxidation appears to have a role in the induction of mitochondrial dysfunction, including in sporadic PD cases [308,309]. Mitochondrial dysfunction in both neurons and microglia themselves can induce microglia activation, resulting in the release of inflammatory cytokines, such as TNF-α and IL-1β, ultimately leading to neuroinflammation and neurodegeneration [310,311]. This self-sustaining cascade of events was postulated two decades ago, although the details of the steps were not evident [312]. Now, it is known that inflammatory cytokines released by microglia disrupt the BBB and induce the expression of adhesion molecules, such as ICAM-1, which facilitate the infiltration of leukocytes, including T cells [310]. Once infiltrated, T cell differentiation is stimulated by different glial cells, but particularly microglia via the release of cytokines. Thus, naïve T cells differentiate into Th1 and Th17 cells, whereas their differentiation into regulatory T cells is suppressed [310,313]. In turn, CD8+ T, Th1, and Th17 cells release inflammatory cytokines, which further promote microglia into an inflammatory and neurotoxic phenotype [310]. IL-17 has been found to increase adhesion molecule expression in microglia [310,314]. Thus, this vicious cycle of glia and T cell reciprocal activation is believed to contribute to the self-sustaining activation of neuroinflammation and neurodegeneration in PD [198,230,310]. 

Although α-synuclein may contribute to the disruption of the BBB [271,315], T cell infiltration appears to precede α-synuclein accumulation in the brain [233]. Interestingly, increased CSF concentrations of ICAM-1 have been associated with increased CSF concentrations of α-synuclein in PD patients [242]. Once α-synuclein begins to accumulate in the SN, the susceptibility to neuronal ferroptosis increases [85,246,260,261,262]. This coincides with the presence of activated glia and T cells and the potentiation of ferroptosis [170,260,261,267,273,310]. Neuronal ferroptosis, in turn, activates T and glial cells, further propagating the inflammatory and degenerative cycle [283,291,292]. In this scenario, ICAM-1 abundance in the SN may facilitate T cell-induced dopaminergic neuronal death and further facilitate interactions amongst glia and T cells [18,35,166,167,168,203].

**Figure 2:**
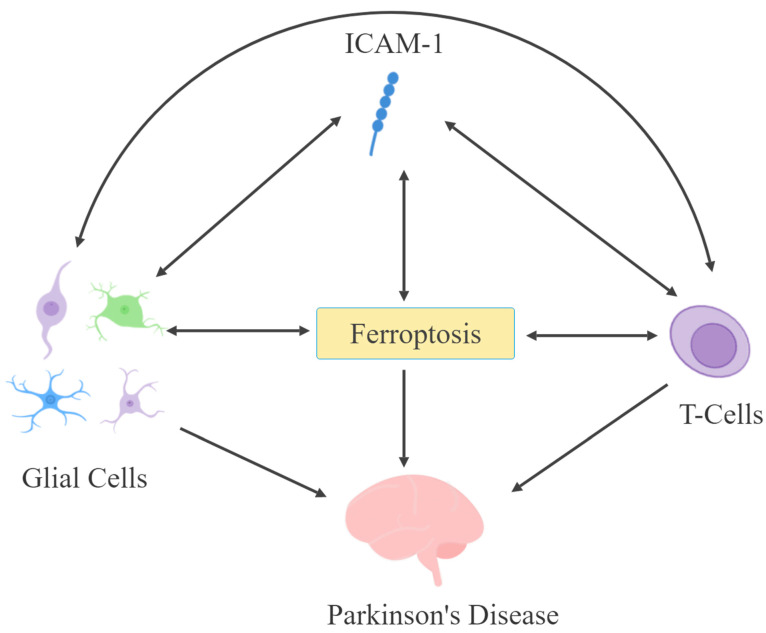
ICAM-1, glial cells, T cells, and ferroptosis may have bidirectional interactions to influence the pathophysiology of Parkinson’s disease.

ICAM-1 expression in astrocytes may be enhanced by α-synuclein [207]. Moreover, ICAM-1-expressing astrocytes are present in the SN and may promote their own expression of ICAM-1 in an ROS- and NF-kB-dependent mechanism [18,183,184,185,186]. However, by far, inflammatory conditions, via the release of cytokines, enhance ICAM-1 expression and can lead to ferroptosis [1,22,23,177,316]. Hence, a vicious cycle may be generated in which ferroptosis would lead to an increase in ICAM-1 expression in endothelial cells, causing the disruption of the BBB and the facilitation of T cell infiltration, leading to further cytokine release, neuroinflammation, and neurodegeneration [85,267,293,298,310]. These direct and indirect interactions between ICAM-1, glia, T cells, and ferroptosis, while elucidating potential mechanisms leading to PD pathophysiology (Figure 2), may also offer novel interventions, as discussed below.

The associations of increased iron accumulation, lipid peroxidation, ROS, ICAM-1, and decreased GSH in the SN during PD further supports that a potential direct ICAM-1–ferroptosis axis exists in this disease [18,85,203,251,257,258,310]. Although circumstantial, various forms of exercise, which was recently advocated as a potential mediator of ferroptosis, has been associated with the decreases in lipid peroxidation, H_2_O_2_, iron accumulation, and sICAM-1 concentrations in patients with PD [317,318,319,320].

## 3. Novel Interventions

The critical need for novel treatment options for PD is well recognized [11,88]. In this regard, ICAM-1 and the ICAM-1–ferroptosis axis could be promising novel targets in PD. The internalization of ICAM-1 in endothelial cells following ICAM-1 antibody binding, and the subsequent recycling of ICAM-1 back into the plasma membrane, has been documented [321]. ICAM-1 antibodies have anti-inflammatory potential by inhibiting leukocyte interactions [321], and have been shown to mitigate PD pathology and symptoms in vivo [231,322]. For example, ICAM-1 antibodies were found to reduce dopaminergic cell death, glial cell activation, gut dysbosis, and behavioral changes in MPTP-treated mice [322]. Likewise, in a previously discussed analysis, LFA-1 and ICAM-1 antibodies were found to decrease immunological and behavioral changes in MPTP-treated mice [231]. Moreover, inhibiting ICAM-1 or LFA-1 has also been found to decrease Treg concentrations in the SN of MPTP-treated mice [323]. Furthermore, catalase-bound ICAM-1 antibodies have been found to inhibit H_2_O_2_ toxicity in endothelial cells during multiple analyses [321,324,325]. The ability for antioxidant enzyme-bound ICAM-1 antibodies to mitigate various neurological conditions, including glial activation, in experimentally induced traumatic brain injury has also been demonstrated in vivo [326,327]. Thus, a number of preclinical studies confirm the utility of ICAM-1 antibodies in mitigating the toxic or neurodegenerative processes.

Moreover, the F(ab’)2 fragment from a murine ICAM-1 antibody was shown to inhibit EAE, and, unlike the murine IgG2a ICAM-1 monoclonal antibody, the F(ab’)2 fragment did not result in the activation of human neutrophils in vitro [188,328]. Although extracellular adherence protein (Eap) of *staphylococcus aureus* interacts with multiple ligands, it binds to ICAM-1, inhibits ICAM-1/LFA-1 interactions, and has been shown to inhibit EAE [329]. The modulation of NG2 protein expression may also represent a viable target for regulating ICAM-1 expression [194]. ICAM-1 is also highly expressed by various cancer cells, and ICAM-1 antibodies conjugated with anticancer drugs have recently been evaluated in vivo as novel approaches to cancer treatment [330,331]. Although these novel approaches in targeting ICAM-1 have yet to be considered within the context of PD, with the execption of ICAM-1 antibodies, the available data suggest potential exploitations of such targets. 

The role of L-dopa in oxidative stress has been debated [332,333,334,335]. Under physiologically relevant conditions, it appears to have antioxidant activity [333,335]. However, elevated concentrations of plasma sICAM-1 were found in stage 1 and 2 in idiopathic PD patients receiving L-dopa, suggesting the particular relevance of ICAM-1 during the early stages of L-dopa treatment [240]. L-dopa-induced dyskinesia has been found to occur concomitantly with an increase in inflammatory cytokines and ROS, and is enhanced in the presence of systemic inflammation in vivo [336,337]. Therefore, the combination of dopamine-enhancing treatments with anti-ICAM-1 treatments would not only address multiple key pathophysiological mechanisms in PD but may also have a synergistic effect with current approaches via mitigating side effects. Together, innovative methods of targeting ICAM-1 and/or the ICAM-1–ferroptosis axis may be a promising option for the treatment and/or mitigation of PD. 

## 4. Conclusions

Recent discoveries indicate a central role for ICAM-1 in PD pathology manifested via its activation of glial cells, as well as the activation and migration of the T cells. Since both glial and T cells are directly linked to ferroptosis, this suggests an indirect connection between ICAM-1 and ferroptosis. ICAM-1 may also have a direct interaction with ferroptosis, which is likely to occur within the context of PD. Although further confirmation of the latter link is needed, collectively, the present knowledge advocates ICAM-1 as a promising target in PD. 

## Data Availability

No new data was created. All citations are availale online.

## Abbreviations

6-OHDA	6-hydroxydopamine
AD	Alzheimer’s disease
ADAM10	a disintegrin and metalloproteinase 10
ADAM17	a disintegrin and metalloproteinase 17
ALOX	arachidonate lipoxygenase
ARE	antioxidant response element
BBB	blood–brain barrier
BDNF	brain-derived neurotrophic factor
CASR	calcium-sensing receptor
CCL2	chemokine ligand 2
CD43	cluster of differentiation 43
CNS	central nervous system
CRP	c-reactive protein
CSF	cerebral spinal fluid
DA	dopamine
DAergic	dopaminergic
DAMPs	damage-associated molecular patterns
DAQ	dopamine quinone
EAE	experimental autoimmune encephalomyelitis
eNOS	endothelial nitric oxide synthase
Fer-1	ferrostatin-1
*FPN1*	ferroportin 1
*FTH1*	ferritin heavy chain 1
GDNF	glial-derived neurotrophic factor
GFAP	glial fibrillary astrocytic protein
GPX4	glutathione peroxidase 4
GSH	glutathione
H_2_O_2_	hydrogen peroxide
*HO-1*	heme oxygenase 1
ICAM1	intercellular adhesion molecule 1
ICAM-1	intercellular adhesion molecule 1
IL-1β	interleukin-1 beta
IL-6	interleukin-6
IFN-γ	interferon gamma
LFA-1	lymphocyte function-associated antigen 1
LPS	lipopolysaccharide
LRP1	low-density lipoprotein receptor-related protein 1
MAC-1	macrophage antigen 1
MDA	malondialdehyde
MMP-2	matrix metalloproteinase 2
MMP-9	matrix metalloproteinase 9
MPTP	1-methyl-4-phenyl-1,2,3,6-tetrahydropyridine
MUC1	mucin 1
nAChRs	nicotinic cholinergic receptors
Nf-kB	nuclear factor kappa B
NG2	nerve/glial antigen 2 (NG2)
NO	nitric oxide
NOX	NADPH oxidase
Nrf2	nuclear factor erythroid 2-related factor 2
OLs	oligodendrocytes
OPCs	oligodendrocyte precursor cells
OS	oxidative stress
PD	Parkinson’s disease
PRRs	pattern-recognition receptors
PUFAs	polyunsaturated fatty acids
ROS	reactive oxygen species
sICAM-1	soluble intercellular adhesion molecule 1
SN	substantia nigra
SNpc	substantia nigra pars compacta
SOD	superoxide dismutase
TfR1	transferrin receptor 1
*TFRCs*	transferrin receptors
TLR2	Toll-like receptor 2
TLR4	Toll-like receptor 4
TLRs	Toll-like receptors
TNF-α	tumor-necrosis factor alpha
TREM2	triggering receptor expressed on myeloid cells-2
VCAM-1	vascular cell adhesion molecule 1
